# Supplementary material for: Biliverdin Reductase B Is a Plasma Biomarker for Intraplaque Hemorrhage and a Predictor of Ischemic Stroke in Patients with Symptomatic Carotid Atherosclerosis
Source: Biomolecules. 2023 May 24;13(6):882. doi: 10.3390/biom13060882 (PMC10296390; doi:10.3390/biom13060882)
Supplement: Supplementary file 1 [file biomolecules-13-00882-s001.zip › biomolecules-2378387-supplementary.pdf]

Supplementary Figures

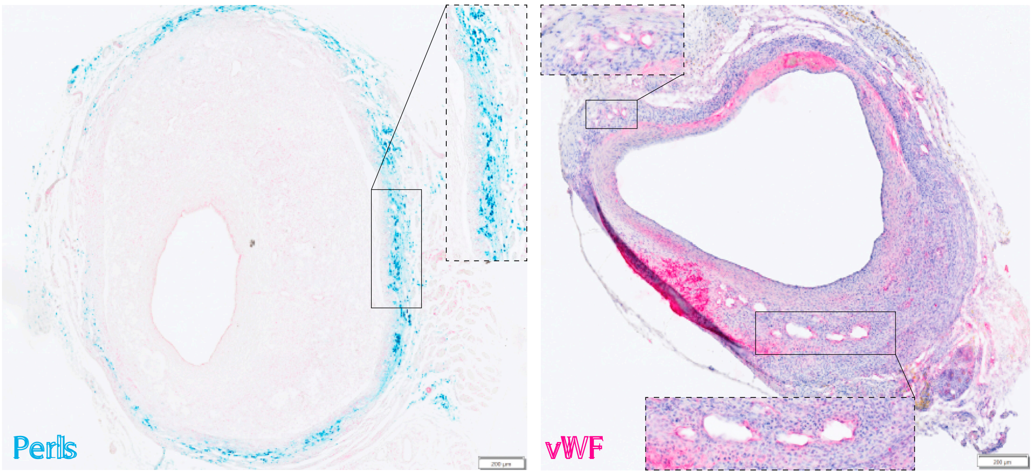

Supplementary Figure S1: Perls and vWF staining in a vein graft mouse model in the control mouse*. Perls staining (blue, left) shows extracellular iron (hemosiderin) deposits from degraded red blood cells. Von Willebrand factor (VWF) (pink, right) stains around neovessels.*

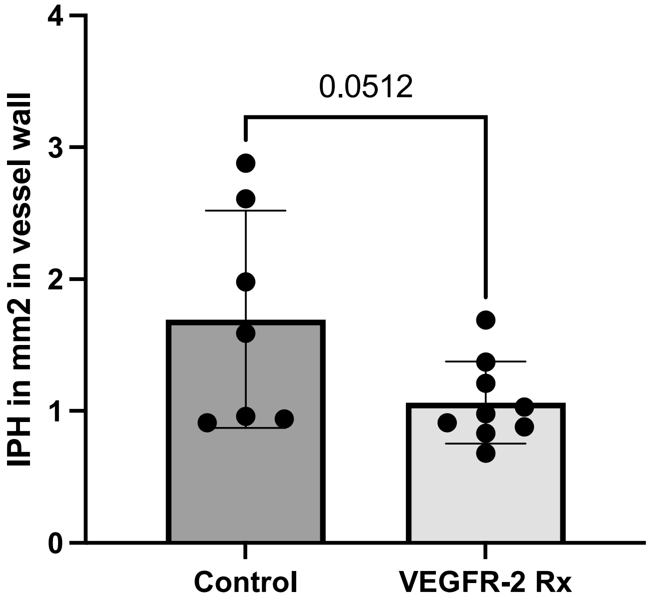

Supplementary Figure S2: IPH assessment in the vessel wall in the vein graft model.
